# Direct IR Absorption Spectra of Propargyl Cation Isolated in Solid Argon

**DOI:** 10.1038/s41598-018-32644-3

**Published:** 2018-09-26

**Authors:** Chih-Hao Chin, Meng-Yeh Lin, Tzu-Ping Huang, Pei-Zhen Wu, Yu-Jong Wu

**Affiliations:** 1 National Synchrotron Radiation Research Center, 101 Hsin-Ann Road, Hsinchu Science Park, Hsinchu, 30076 Taiwan; 2 Department of Applied Chemistry, National Chiao Tung University, 1001, Ta-Hsueh Road, Hsinchu, 30010 Taiwan

## Abstract

The direct infrared (IR) absorption spectra of propargyl cations were recorded. These cations were generated via the electron bombardment of a propyne/Ar matrix sample during matrix deposition. Secondary photolysis with selected ultraviolet (UV) light was used for grouping the observed bands of various products. The band assignment of the propargyl cation in solid Ar was performed according by referring to the previous infrared photodissociation (IRPD) and velocity-map imaging photoelectron (VMI-PE) data, and via theoretical predictions of the anharmonic vibrational wavenumbers, band intensities, and deuterium-substituted isotopic ratios. Almost all the IR active bands with an observable intensity were recorded and the ν_11_ mode was reported for the first time.

## Introduction

C_3_H_3_^+^ ions are important in many diverse applications and fields, such as flame and combustion processes, and planetary and interstellar chemistry^1–5^. The two most stable isomers of C_3_H_3_^+^, cyclopropenyl cations (c-C_3_H_3_^+^) and propargyl cations (H_2_C_3_H^+^), have received much attention in the past decades. Both are commonly observed fragments in the combustion of hydrocarbons and are proposed as reactants in the synthesis of cumulenic and cyclic C_3_H_2_ in the interstellar medium through ion–molecule reactions^6,7^. They have also been detected in the tail of Halley’s comet^8^. In addition, because these cations and their neutral counterparts are the smallest π-conjugated hydrocarbon systems, their molecular structures, reactivities, and spectroscopic parameters have been extensively investigated both theoretically and experimentally^9–15^.

Theoretical works have indicated that the energy of planar H_2_C_3_H^+^ (C_2v_) is ~28 kcal mol^−1^ greater than that of cyclic planar c-C_3_H_3_^+^ (D_3h_)^16,17^. The vibrational fundamentals of both cations complexed with several ligands recorded via infrared photodissociation (IRPD) have been reported, and with the complexation with Ar, there are obvious shifts of the C-H stretching bands, but the C-C stretches are unaffected^11,18,19^. The infrared (IR) spectra of both cations were also recorded in neon matrices at 5 K, but only the C≡C stretching mode (ν_3_) of H_2_C_3_H^+^ and the C-H asymmetric stretching mode (ν_4_) of c-C_3_H_3_^+^ were observed^20^. Recently, Gao *et al*. combined the vacuum ultraviolet (VUV) lasers and velocity-map imaging (VMI) techniques to obtain the vibrationally resolved photoelectron spectrum (PE) of gaseous H_2_C_3_H radicals and determined the C≡C stretching (ν_3_), C-C stretching (ν_5_), and out-of-plane bending (ν_7_) modes of the ground-state H_2_C_3_H^+^^21^. A discrepancy was observed in the band position of the C-C stretching mode by 102 cm^−1^ when the IRPD and VUV-VMI-PE results were compared. Later, Botschwina *et al*.^17^, performed explicitly correlated coupled cluster calculations for C_3_H_3_^+^ species and confirmed the assignment of the C-C stretching mode of H_2_CCCH^+^ determined by Gao *et al*. They also suggested that the assignment of the asymmetric CH stretching mode (ν_4_) of c-C_3_H_3_^+^-Ar at 3182 cm^−1^ reported by Ricks *et al*.^18^ should be the combination band (ν_3_ + ν_5_) of H_2_C_3_H^+^-Ar. In addition, the *A* ^1^A_1_ ← *X* ^1^A_1_ electronic transition of H_2_CCCH^+^ in the neon matrix, and that tagged with Ne and N_2_ were measured in the spectral region of 230–270 nm^22^. In contrast, the electronic transitions of c-C_3_H_3_^+^ are expected to appear at less than 200 nm, but no experimental values for the same have been reported in any conditions^20^.

Although the spectroscopic information of H_2_C_3_H^+^ has been obtained by various experimental techniques, there is lack of clarity with regard to its vibrational structures. The IRPD method could not provide the true (relative) IR intensities of species, and laser frequencies are typically useful above 1000 cm^−1^ which limit the probing spectral region^18^. The VUV-VMI-PE method can be used to obtain information about the low-frequency vibrational modes, but the selection rule of photoelectron spectroscopy differs from that of IR absorption spectroscopy^21^. We employed a “clean” method of matrix isolation for investigating the IR spectra of cations of interest using electron bombardment of an Ar matrix containing a small proportion of neutral counterparts during matrix deposition. We demonstrated the advantages of this method with ethylene and allene cations^23,24^. Our method can be used to obtain IR spectra with a high resolution, the true IR intensities, and a wider spectral coverage of cations of interest.

In our previous works, we produced allene cations (H_2_C_3_H_2_^+^) in solid Ar via electron bombardment (200 eV) of an allene/Ar matrix sample during matrix deposition^24^. Subsequently, we irradiated the matrix sample with 365-nm light, which resulted in isomerization from allene cations to propyne cations (H_3_C_3_H^+^) in the solid Ar. We further characterized the IR signatures of the allene and propyne cations, and the IR absorption spectrum of the propyne cations was reported for the first time. Forney *et al*. used excess excited Ne atoms to enable collisions with allene or propyne, followed by co-deposition at 5 K; they observed similar products including H_2_C_3_H_2_^+^, H_2_C_3_H^−^, HC_3_H, and C_3_, in both cases^25^. They concluded that propyne cations might predominantly isomerize to the more stable allene cations in the experiments^25^. In this work, we subjected propyne/Ar matrix samples to electron bombardment at 200 or 2000 eV, followed by deposition at 8 K. H_2_C_3_H_2_^+^ cations were found to be dominant for low-energy bombardment, similar to the case in previous allene experiments. This suggests that the loss of H atoms from the two precursors might be inhibited due to the rapid excess energy quenching after bombardment, while several H-degradation species including H_2_C_3_H^+^ were formed in the high-energy bombardment experiment.

## Results and Discussion

### IR absorption spectra of matrix samples in natural isotopic abundance

**Figure 1:**
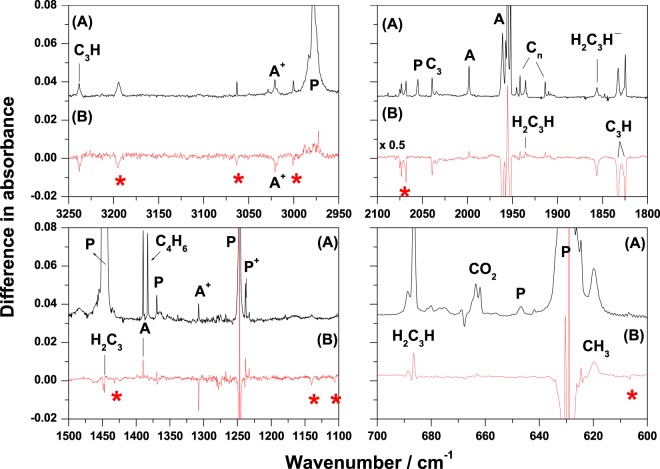
(**A**) Partial IR spectrum of a propyne/Ar (1/500) matrix sample bombarded with 2000-eV electrons at 8 K (absorption multiplies 0.2) and (**B**) IR difference spectrum of sample in (**A**) upon photolysis with 385-nm light for 1 h. A: H_2_C_3_H_2_, P: H_3_C_3_H, A^+^: H_2_C_3_H_2_^+^, P^+^: H_3_C_3_H^+^, *: H_2_C_3_H^+^, C_n_: carbon clusters.

We subjected the propyne/Ar matrix sample to bombardment with 200-eV electrons and recorded the corresponding IR spectra, as shown in Fig. S1 in supplementary information (SI). The products and observed absorptions are summarized in Table 1. Compared to the previous studies on the electron bombardment of allene^24^, the observed products are similar and no propyne cations were observed. The allene neutrals and cations were the main products, indicating that the fragmentations of the precursor were less prominent upon low-energy bombardment. The IR spectra of both the neutral and cationic allene and propyne species have been well studied, and therefore we increased the electron energy to 2000 eV to increase the efficiency of the production of fragments from the precursor. Partial IR spectra of the electron-bombarded propyne/Ar matrix sample are shown in Fig. 1(A) and the observed bands and assignments are summarized in Table 1.

**Table 1:** Observed species generated from bombardment of H_3_C_3_H/Ar matrix sample with 200-eV and 2000-eV electrons and their photolytic behaviors.

Species	Observed band positions/cm^−1^	Ref.	385 nm	160 nm
HC_3_H^a^	547.6, 3266.2 (3262.9)^b^	^25^	↑^c^	—^c^
H_3_C_3_H^+^	583.2 (581.1), 1243.0	^24^	↑	↓
CH_3_	619.9	^33^	↑	↓
H_2_C_3_H^a^	686.5, 1061.8, 1935.4, 3028.3, 3310.0	^25^	↑	↓
HCCH^a^	737.2, 3285.7 (3287.9), 3293.3	^34^	↑	—
C_6_H_4_	740.7 (743.7), 1008.7, 1040.9,	^35^	↑	↓
H_2_C_3_H_2_^+a^	791.4, 874.4, 1307.3, 2929.0, 3020.8	^25^	↓	↓
H_2_CC(H)CH_2_	800.9, 1179.4, 3018.9	^36^	↓	↑
H_2_C_3_H_2_^a^	837.7, 996.2, 1390.6, 1955.3, 1998.4, 3000.1	^24^	↑	↓
Ar_2_H^+a^	903.4	^24^	↓	↓
C_4_H_6_^a^	910.8, 1016.8, 1382.9	^37^	—	↓
H_2_C_3_	999.2 (1003.0), 1446.8 (1448.6)	^38^	↓	—
HC_3_^a^	1824.8 (1832.6), 3238.3	^38^	↓	↑
H_2_C_3_H^−a^	1856.4	^25^	↓	—
C_n_	1913.8, 1941.8, 1945.8		↑	—
C_3_	2039.2 (2034.8)	^25^	↓	↑
H_2_C_3_H^+^	606.8, 1105.2, 1140.6, 1433.2, 2075.2 (multiplet), 3000.6, 3063.4, 3195.3	TW	↓	↓

^a^Species also observed in experiments on electron bombardment with 200-eV electrons.

^b^Bands in minor matrix sites.

^c^Symbols indicate band intensity increase (↑), decrease (↓), and no change (—) upon photolysis.

Besides the species observed in the 200-eV experiments, more types of C_3_H_n_ species were formed in observable concentrations. For example, another C_3_H_2_ form, H_2_C_3_, was observed with IR bands at 999.2 and 1446.8 cm^−1^ corresponding to the H_2_CC OPLA and CH_2_ scissoring modes, respectively. The absorptions of HC_3_ and C_3_ were also observed to be moderately intense. This indicates that increasing the bombardment energy of electrons is useful for promoting the removal of H atoms from the precursors. In addition, we observed larger species, such as C_6_H_4_ (benzyne) and C_4_H_6_ (1,3-butadiene), which may be a result of the recombination reactions of the small fragments. We also confirmed the formation of allyl radicals (H_2_CCHCH_2_) based on the IR bands at 800.9, 1179.4, and 3018.9 cm^−1^. The formation of the allyl radicals may be due to the H-addition reaction of the allene neutrals.

Despite the reported information of the IR spectra of the above-mentioned species in solid matrices, which are easily assigned in our study, there are several unknown bands. Therefore, we performed further experiments on the photolysis of the matrix sample with 385- and 160-nm light to classify the unknown bands. The photolytic behaviors of the observed products are summarized in Table 1. Figure 1(B) shows the IR difference spectrum of the electron-bombarded matrix sample irradiated with 385-nm light, in which a positive value indicates a species produced after irradiation, whereas a negative value indicates the destruction of a species. The IR difference spectrum of the matrix sample further irradiated with 160-nm light is shown in Fig. S2 in SI. A set of unknown bands at 606.8, 1105.2, 1140.6, 1433.2, 2075.2 (multiplet), 3000.6, 3063.4, and 3195.3 cm^−1^, indicated by asterisks (*) in Fig. 1(B), showed a decrease in intensity upon photolysis at both wavelengths. The positions of this set of bands correlate well with those of most bands of gaseous H_2_C_3_H^+^ determined via IRPD measurements^18^ and with the only reported IR band at 2080 cm^−1^ for H_2_C_3_H^+^ in solid neon^20^, as shown in Table 2. Furthermore, the observed photolytic behavior of the * bands is similar to that of H_2_C_3_H_2_^+^ and Ar_2_H^+^ cations suggesting it might be a charged species. The decrease in the cationic species upon irradiation with 385-nm light has been demonstrated in many previous studies^23,24,26^ and this may be because the UV light caused the slowly diffusing trapped electrons to recombine with the cations. The increase in the H_3_C_3_H^+^ cations upon irradiation with 385-nm light in this study is due to the isomerization of H_2_C_3_H_2_^+^^24^. In contrast, the intensities of the other unknown bands at 1913.8, 1941.8, and 1945.8 cm^−1^ increased upon irradiation with 385-nm light, but showed no change with further irradiation of 160-nm light. In addition, there was no correlation to each other at the photolysis stage. These bands are the result of the absorption of typical multiple CC stretches and may correspond to carbon-chain species. Deuterium-isotopic experiments and quantum-chemical calculations were also performed to obtain information for assigning these bands.

**Table 2:** Comparison of experimental and predicted anharmonic vibrational wavenumbers of propargyl cations. Predicted IR intensities are listed in parentheses.

Mode	Sym	B3LYP /aug-cc-pVTZ	CCSD(T*)-F12a/VTZ-F12	IRPD	PE	Matrix
*v*_1_	A_1_	3226.4 (115)^a^	3236 (112)a	3238		3195.3 (28)^b^
*v*_2_	A_1_	3007.1 (30)	2990 (29)	3004		3000.6 (10)
*v*_3_	A_1_	2088.1 (370)	2080 (371)	2077	2086	2075.2^c^ (100)
*v*_4_	A_1_	1458.3 (9)	1446 (12)	1445		1433.2 (6)
*v*_5_	A_1_	1137.2 (25)	1123 (19)	1222	1120	1140.6 (13)
*v*_6_	B_1_	1112.4 (10)	1099	1111		1105.2 (6)
*v*_7_	B_1_	904.4 (5)	872		858	
*v*_8_	B_1_	279.3 (25)	264			
*v*_9_	B_2_	3043.0 (45)	3080	3093		3063.4 (22)
*v*_10_	B_2_	1012.4 (1)	1017			
*v*_11_	B_2_	625.7 (58)	615			606.8 (15)
*v*_12_	B_3_	331.2 (15)	298			
Ref		This work	^17^	^18^	^21^	This work

^a^The unit of the calculated IR band intensity shown in the parentheses is km mol^−1^.

^b^The relative band intensities of the observed IR bands of H_2_CCCH^+^ are shown in the parentheses.

^c^This band was reported at 2080 cm^−1^ for solid Ne^20^.

### IR absorption spectra of deuterium-substituted samples

**Figure 2:**
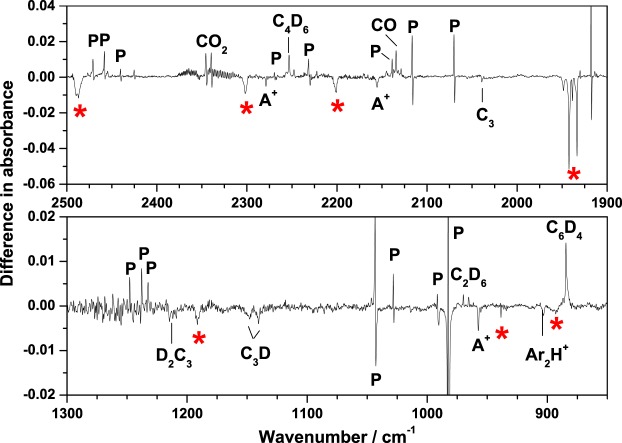
Partial IR spectrum of electron-bombarded deuterium-substituted D_3_C_3_D/Ar (1/500) matrix sample upon photolysis with 385-nm light at 8 K. P: D_3_C_3_D, A^+^: D_2_C_3_D_2_^+^, and *: D_2_C_3_D^+^.

To confirm the preliminary assignments of the unknown bands, the fully deuterium-substituted propyne (D_3_C_3_D) was used to determine the isotopic shifts of the corresponding bands. Experiments on the electron bombardment of D_3_C_3_D/Ar (1/500) were performed. The representative IR difference spectrum in the range of 850−1300 and 1900−2500 cm^−1^ for irradiation at 385 nm is shown in Fig. 2. The lines observed for D_2_C_3_D^+^ and H_2_C_3_H^+^ are listed in Table 3 with the vibrational wavenumbers and isotopic ratios. Based on the observed photolytic behavior, relative band intensity, and expected D-isotopic shift ratios of the vibrational modes, the set of unknown bands at 1105.2, 1140.6, 1433.2, 2075.2 (multiplet), 3000.6, 3063.4, and 3195.3 cm^−1^ were found to shift to 891.3, 938.7, 1191.7, 1942.1 (multiplet), 2201.0, 2301.9, and 2487.3 cm^−1^, respectively, as indicated by the asterisk (*) in Fig. 2. The band corresponding to that at 606.8 cm^−1^ observed in natural abundance was not observed in the D-isotopic experiment. It may have shifted to the spectral region beyond detection.

**Table 3:** Comparison of D-isotopic ratios of experimental and theoretical vibrational wavenumbers of propargyl cations.

Mode	Line position/cm^−1^		Isotopic ratio^a^		
	H_2_C_3_H^+^	D_2_C_3_D^+^	Prediction^b^	Ne matrix	Ar matrix
*v*_1_	3195.3	2487.3	0.7768		0.7784
*v*_2_	3000.6	2201.0	0.7304		0.7335
*v*_3_	2075.2	1942.1	0.9352	0.9399	0.9368
*v*_4_	1433.2	1191.7	0.8327		0.8315
*v*_5_	1140.6	938.7	0.8315		0.8330
*v*_6_	1105.2	891.3	0.8012		0.8065
*v*_9_	3063.4	2301.9	0.7458		0.7514
*v*_11_	606.8	—	0.7765		—
Ref	This work	This work	This work	^20^	This work

^a^The ratio of the wavenumber of the deuterium isotopic species to that of H_2_C_3_H^+^.

^b^Theoretical values predicted using B3LYP/aug-cc-pVTZ.

In contrast, there was no shift in the unassigned bands at 1913.8, 1941.8, and 1945.8 cm^−1^ in the natural isotopic abundance sample during the D-isotopic substitution, suggesting that the carriers of these bands contain no H atoms; rather, they may be neutral and/or ionic carbon clusters (C_n_).

### Quantum chemical calculations and band assignments

We performed quantum-chemical calculations with the B3LYP/aug-cc-pVTZ basis set to determine the stable structure of H_2_C_3_H^+^ (C_2v_). The structural parameters for the propargyl cation are shown in Fig. S3. Information about the mode symmetry, vibrational motion, anharmonic vibrational frequencies, and IR intensities of the propargyl cation is summarized in Table 2 and Fig. S4, along with the values in the literature for comparison. Compared to the anharmonic vibrational wavenumbers of H_2_C_3_H^+^ predicted using CCSD(T*)-F12a^17^, the deviation is less than 3.5% for all vibrational modes, except for the low vibrational frequency mode for the CCC bending (ν_12_). Our calculated band intensities are also consistent with the high-level theoretical work^17^.

Compared to our experimental observations, almost all the features predicted with an observable IR intensity in the probed spectral region were observed. For example, the C-H stretching modes predicted at 3226.4, 3007.1, and 3043.0 cm^−1^ with a moderate intensity were observed at 3195.3, 3000.6, and 3063.4 cm^−1^, respectively. The most intense mode of the C≡C stretch predicted at 2088.1 cm^−1^ fitted well with the strong absorption observed at 2075.2 cm^−1^ (multiplet). The other weak absorption bands observed at 1433.2, 1140.6, 1105.2, and 606.8 cm^−1^ were also consistent with the predictions in terms of both the band positions and band intensities. Table 3 lists the vibrational wavenumbers and isotopic ratios predicted using the B3LYP/aug-cc-pVTZ level of theory along with the experimental observations. The observed deuterium isotopic ratios for D_2_C_3_D^+^ agreed with the theoretically predicted values with the largest deviation of only ~0.53% for the ν_6_ mode. The findings of the deuterium isotopic experiments clearly support the previous assignments of the * bands to the propargyl cation.

There was a discrepancy between the IRPD and VMI-PE results of the vibrational wavenumbers of the ν_5_ mode of the propargyl cation by 102 cm^−1 ^^18,21^. In our study, the absorption was determined to be at 1140.6 cm^−1^ for the ν_5_ mode, close to the VMI-PE result^21^. Furthermore, the CCH bending (ν_11_) mode of the propargyl cation was determined for the first time. Although the cyclopropenyl cation is more stable than the propargyl cation, we did not observe any bands correlated to the absorption of the cyclopropenyl cation. This may be due to the following reasons: (i) the linear precursor, H_3_C_3_H, was used to generate the C_3_H_3_^+^ species, and (ii) H_2_C_3_H^+^ was formed and subsequently trapped in the solid Ar, which may have inhibited further isomerization.

## Conclusion

Propargyl cations were generated from bombardment of a mixture of propyne and Ar with 2000-eV electrons during deposition at 8 K. The lines of the propargyl cation were diminished via irradiation of the matrix with UV light at 385 and 160 nm. The assignment of vibrational bands was based on a comparison with results from previous reports and anharmonic vibrational wavenumbers, IR intensities, and deuterium-substituted isotopic ratios predicted for the propargyl cation. This study confirmed the assignment of the ν_5_ mode at 1140.6 cm^−1^ and recorded the IR absorption of the ν_11_ mode for the first time. The “true” experimental relative IR intensities of the observed vibrational modes of the cation were also determined.

## Experimental and Theoretical Methods

The experimental setup has been described previously^27,28^. The IR absorption spectra covering the spectral range of 500–4000 cm^−1^ were recorded with an interferometric spectrometer (Bruker v80) equipped with a KBr beam splitter and a Hg–Cd–Te detector cooled to 77 K. Typically, 400 scans at a resolution of 0.5 cm^−1^ were recorded at each stage of the experiment.

The cations were produced via the electron bombardment of a gaseous Ar sample containing a small proportion of propyne during the deposition stage. A 200- or 2000-eV electron beam at a current of 0.3 mA was generated with an electron gun (Kimball Physics, Model EFG-7). A gaseous mixture of H_3_CCCH/Ar (1/500) was deposited over a period of 4 h at a flow rate of 5–8 mmol h^−1^. Experiments with a deuterium-substituted sample, D_3_CCCD/Ar (1/500), were conducted under the same conditions. Photolysis experiments were performed with synchrotron radiation at BL03 of NSRRC (~5 mW at 160 nm), and with a light-emitting diode (bandwidth ~10 nm, 350 mW at 385 nm). Ar (99.9999%, Scott Specialty Gases), H_3_CCCH (99.5%, Aldrich), and D_3_CCCD (deuterium ~99%, Aldrich) were used without further purification, except for a freeze–pump–thaw procedure at 77 K.

The energy, equilibrium structure, anharmonic vibrational wavenumbers, and IR intensities of the propargyl cation were calculated using the Gaussian 09 program^29^ at the B3LYP/aug-cc-pVTZ level of theory^30–32^.

## Electronic supplementary material

Supplementary information
