# Supplementary material for: Direct IR Absorption Spectra of Propargyl Cation Isolated in Solid Argon
Source: Sci Rep. 2018 Sep 26;8:14392. doi: 10.1038/s41598-018-32644-3 (PMC6158168; doi:10.1038/s41598-018-32644-3)
Supplement: Supplementary file 1 — Supplementary information [file 41598_2018_32644_MOESM1_ESM.doc]

**Supplementary Information**

Direct IR Absorption Spectra of Propargyl Cation Isolated

in Solid Argon

Chih-Hao Chin,^1^ Meng-Yeh Lin,^1^ Tzu-Ping Huang,^1^ Pei-Zhen Wu,^1^

and Yu-Jong Wu^1,2^,*

^1^National Synchrotron Radiation Research Center, 101 Hsin-Ann Road, Hsinchu Science Park, Hsinchu 30076, Taiwan

^2^Department of Applied Chemistry, National Chiao Tung University, 1001, Ta-Hsueh Road, Hsinchu 30010, Taiwan

*E-mail: yjwu@nsrrc.org.tw

**Figure S1** Partial IR spectrum of the matrix sample (H_3_CCCH/Ar = 1/500) bombarded with 200-eV electrons. Assignments of bands are indicated. A: allene, A^+^: allene cation, and P: propyne.

**Figure S2** Difference IR spectrum of the electron bombarded matrix sample in Fig. 1(B) further irradiated with 160 nm for 1 h. Assignments of bands are indicated. A: allene, A^+^: allene cation, and P: propyne, P^+^: propyne cation.

**Figure S3** Comparison of predicted structural parameters of the propargyl cation (C_2v_).

**Figure S4** Predicted vibrational modes of propargyl cation (C_2v_) at the B3LYP/aug-cc-pVTZ level of theory.
